# Evolution of Feeding Shapes Swimming Kinematics of Barnacle Naupliar Larvae: A Comparison between Trophic Modes

**DOI:** 10.1093/iob/obaa011

**Published:** 2020-04-17

**Authors:** J Y Wong, B K K Chan, K Y K Chan

**Affiliations:** 1 Department of Life Science, National Taiwan Normal University, Taipei 11677, Taiwan; 2 Biodiversity Program, Taiwan International Graduate Program, Academia Sinica, Taipei 11529, Taiwan; 3 Biodiversity Research Center, Academia Sinica, Taipei 11529, Taiwan; 4 Biology Department, Swarthmore College, Swarthmore, PA 19081, USA

## Abstract

A central goal in evolutionary biology is connecting morphological features with ecological functions. For marine invertebrate larvae, appendage movement determines locomotion, feeding, and predator avoidance ability. Barnacle larvae are morphologically diverse, and the morphology of non-feeding lecithotrophic nauplii are distinct from those that are planktotrophic. Lecithotrophic larvae have a more globular body shape and simplified appendages when compared with planktotrophs. However, little is known about whether and how such morphological changes affect kinematics, hydrodynamics, and ecological functions. Here, we compared the nauplii kinematics and hydrodynamics of a lecithotrophic Rhizocephalan species, *Polyascus planus*, against that of the planktotrophic nauplii of an intertidal barnacle, *Tetraclita japonica*. High-speed, micro-particle image velocimetry analysis showed that the *Polyascus* nauplii swam faster and had higher amplitude and more synchronous appendage beating than the *Tetraclita* nauplii. This fast swimming was accompanied by a faster attenuation of induced flow with distance, suggesting reduced predation risk. *Tetraclita* nauplii had more efficient per beat cycles with less backward displacement during the recovery stroke. This “anchoring effect” resulted from the anti-phase beating of appendages. This movement, together with a high-drag body form, likely helps direct the suction flow toward the ventral food capturing area. In sum, the tradeoff between swimming speed and predation risks may have been an important factor in the evolution of the observed larval forms.

## Synopsis

**(Chinese)** 瞭解形態特徵與生態功能的聯繫是演化生物學的重要課題。對於海洋無脊椎動物的幼體, 牠們附肢的運動對其游動, 進食和避免捕食者的能力有決定性的影響。藤壺無節幼體形態多變, 其中卵黃食性的幼體和以浮游生物為食的幼體形態迴異。卵黃食性的無節幼體體型呈球狀,附肢較簡化。不過, 我們對這些形態差異如何影響運動學, 流體動力學以及生態功能的所知之甚少。我們比較了以浮游生物為食的日本笠藤壺(*Tetraclita japonica*) 無節幼體和扁平多囊蟹奴(*Polyascus planus*)卵黃食性的無節幼體的運動學和流體動力學。高速粒子圖像測速分析顯示, 與以浮游生物為食的幼體相比, 卵黃食性的幼體游動更快, 附肢振幅更高, 肢體拍打更同步。卵黃食性的幼體不但游速較高, 其游泳造成的水流衰弱亦較快, 有助降低被捕食者偵測到的風險。日本笠藤壺的幼體雖然游得較慢, 但身體在主要游泳附肢回收到起始位置時有較小的向後位移。這種“錨定效應”是由第一觸角與另兩對附肢的反相位搏動而引起的。這種附肢運動加上高水阻的身體形態或能幫助幼體將用於捕食的抽吸流引向腹側的食物捕獲區域。簡言之, 游泳速度和被捕食風險之間的取捨可能是影響幼體形態演變的重要因素。

## Introduction

Nauplius is a homologous developmental stage shared by all crustaceans, and the free-living form of nauplius has persisted in most lineages (Williams 1994b) but see Scholtz (2000). Despite being a conserved larval stage, the body forms of free-living nauplii are diverse (Dahms et al. 2006; Martin et al. 2014), and differences in swimming behaviors have been reported (Gauld 1959; Moyse 1984). It was posited that lability in naupliar phenotypes, especially that of behavior, allows diverse functions to evolve, which in turn contribute to the persistence of the nauplius during the adaptive radiation of crustaceans (Williams 1994a). However, few data are available on the relationship between naupliar morphology and kinematics, and on how phenotypic differences translate to functional performance by changing the nauplii’s interactions with the surrounding fluid.

Swimming kinematics and/or hydrodynamics of nauplii have been previously described, mainly for copepods (Johnson et al. 2011; Borg et al. 2012; Kiørboe et al. 2014; Wadhwa et al. 2014; Lenz et al. 2015). And yet, the studied copepod nauplii represent only a fraction of known naupliar forms. A striking example of diversity in naupliar forms can be found among barnacle (Cirripedia) nauplii. They are easily distinguished from other crustacean nauplii by the presence of a pair of frontal horns, which are unique for barnacles (Høeg and Møller 2006). The presence of frontal horns or the less streamlined overall naupliar forms of barnacles was thought to be costly for locomotion, but may be beneficial for suspension feeding (Moyse 1984; Emlet and Strathman 1985). Comparative study on barnacle naupliar forms supports this functional tradeoff: common planktotrophic nauplii have relatively longer frontal horns and tail spines than lecithotrophic nauplii that do not feed (Wong et al. 2018). However, without empirical data on how lecithotrophic nauplii perform, inference on such a morphology–function link still lacks mechanistic insight (Koehl 1996).

Planktotrophic barnacle nauplii are “current feeders.” They are capable of cruising through water and generating feeding currents at the same time (Lochhead 1936). When locomotion is tightly linked with feeding, a compromise between the two functions is highly likely (Strathmann and Grünbaum 2006). For instance, an optimized mode of propulsion in nauplii is to paddle all three appendages pairs radially to push themselves forward. However, such movement would lead to food particles being pushed away from the body, compromising feeding. Another example of a tradeoff is that feeding currents spanning a larger area will entrain more food particles. And yet, the associated fluid disturbance will pose a higher predation risk by rheotatic predators (Kiørboe et al. 2010, 2014). In sum, not only is locomotory performance constrained by the need to feed, but also the need to retain stealth for protection from predators.

Lecithotrophic nauplii have evolved a few times within Cirripedia and can be found in all three superorders (Martin et al. 2014). Most of them are found in parasitic barnacles or are associated with adaptation to oligotrophic habitats for larvae. Rhizocephala, the superorder with barnacles all specialized in parasitism, have only lecithotrophic nauplii (Høeg 1995). We hypothesize that swimming of lecithotrophic rhizocephalan nauplii, which are released from the constraint of feeding, will display kinematic characteristics that support the model of optimized nauplius swimming (Takagi 2015), and hydrodynamic signals that minimize predation risk.

Here, we compared kinematics and hydrodynamics of the nauplii of the rhizocephalan species *Polyascus planus*, which are internal parasites on grapsid crabs, against those of the free living intertidal barnacle *Tetraclita japonica*. We focused on the performance related to three major sources of selection pressure: locomotion, predation risk, and feeding. We specifically compared the proficiency (normalized velocity) and efficiency (forward: backward displacement ratio) of swimming, the spatial attenuation of flow signal to predators, and flux of suction current generated during the recovery stroke. We also compared swimming kinematics which likely lead to these differences in performance.

## Methods

### Collection of nauplii

Adults of *T. japonica* were collected from the rocky intertidal in Clear Water Bay, Hong Kong (22°20′22″N 114°16′E). After collection, egg masses were dissected from the mantle cavity of *T. japonica* and maintained in aerated filtered seawater (25°C, 33 psu) until nauplii hatched. Host crabs of *P. planus* (*Grapsus albolineatus* and *Pachygrapsus crassipes*) with visible externa were hand caught from the rocky intertidal at Badouzi, NE Taiwan (25°08′50″N 121°47′40″E), and reared until release of nauplii from the externa. All hatched nauplii were transferred to fresh filtered seawater (25°C, 33 psu), and reared to stage II for video observations. Nauplii morphometrics data were gathered through digital microscopy and are presented in Table 1.

**Table 1:** Morphometrics, swimming performance, kinematics, and hydrodynamics comparisons of the barnacle nauplii

	*Polyascus* (lecithotrophic)		*Tetraclita* (planktotrophic)	
Morphometrics				
Carapace length (µm)	265.0 ± 7.4		447.4 ± 16.8	
Carapace width (µm)	173.3 ± 5.6		242.0 ± 10.8	
Carapace height (µm)	112; 115		121; 146	
Carapace area (mm^2^)	0.0326 ± 0.0006		0.0566 ± 0.0016	
Swimming performance				
Speed (mm s^−1^)	**7.7 ± 0.4**		**4.5 ± 0.5**	
Speed (body length s^−1^)	**29.2 ± 1.2**		**10.0 ± 1.1**	
Forward:backward displacement ratio	**6.2 ± 0.2**		**9.3 ± 0.7**	
Kinematics				
Frequency (Hz)	**35.7 ± 2.1**		**11.4 ± 0.7**	
Amplitude (°)				
ant1	50.8 ± 2.0		48.9 ± 4.9	
ant2	94.8 ± 1.2		90.5 ± 3.3	
mand	**79.0 ± 3.8**		**56.5 ± 1.4**	
% in phase				
ant1–ant2	**67 ± 3**		**52 ± 5**	
ant1–mand	40 ± 5		28 ± 4	
ant2–mand	69 ± 3		71 ± 4	

	Mid-power stroke	Mid-recovery stroke	Mid-power stroke	Mid-recovery stroke

Angular speed (°/ms)				
ant1	**6.5 ± 0.5**	**6.3 ± 0.5**	**1.9 ± 0.3**	**1.9 ± 0.3**
ant2	**11.8 ± 0.3**	**10.4 ± 0.4**	**3.5 ± 1.5**	**3.3 ± 0.4**
mand	**13.3 ± 0.9**	**9.7 ± 0.8**	**3.0 ± 0.3**	**2.3 ± 0.4**
Angular separation (°)				
ant1–ant2	**53.1 ± 3.5**	**18.3 ± 2.7**	**77.2 ± 4.8**	**32.2 ± 2.7**
ant1–mand	**117.0 ± 2.5**	**26.1 ± 3.0**	**146.4 ± 1.9**	**59.7 ± 4.3**
ant2–mand	63.9 ± 2.9	**7.8 ± 0.4**	69.2 ± 0.4	**27.5 ± 3.7**
Hydrodynamics				
Reynolds number	2.20 ± 0.11		2.13 ± 0.20	
Circulation at the end of the power stroke (mm^2^ s^−1^)	−2.33 ± 0.16		−2.13 ± 0.15	
Area of influence (mm^2^)	**0.50 ± 0.02**		**1.08 ± 0.02**	
Estimated strongest relative flux (mm^2^ s^−1^)				
Dorsal view	−1.50 ± 0.05		−1.09 ± 0.16	
Lateral view	−01.61, −0.95		−0.57, −0.87	

Values are mean ± SE (*n *=* *5) except for carapace height and flux that were calculated from lateral view (*n *=* *2). % in phase compares percentage of pairs of appendages moving in the same direction. Values with statistically significant differences between taxa are bolded (*P*-value < 0.05, permutational *T*-test run with 9999 permutations). ant1, antennule; ant2, antenna; mand, mandible.

### Video acquisition

A custom-made glass cuvette (25 × 75 × 5 mm) was used as a recording chamber and held inside a dark room with temperature maintained at 25°C. An external tank with larger volume of water (400 mL) was used to buffer small temperature fluctuations. A high-speed camera (FastCam Mini UX100, Photron Ltd.) fitted with a bellows and a 60 mm focal length lens was used to video record swimming nauplii. Illumination was achieved with an array of white LEDs. Video acquisition was controlled with PFV software (Photron Ltd.) and recorded with a resolution of 1280 × 1024 pixels at 2000 frames s^−1^. Microalgae (*Isochrysis galbana*) and neutrally buoyant micro-plastic beads (2.32 µm in diameter, Spherotech Inc.) were used as seeding particles to trace the fluid flow around *T. japonica* and *P. planus* nauplii, respectively. About 30 individuals were used in each video session. The nauplii were not tethered, so successful recording depended on nauplii passing the field of view on the right focal plane (see details in the Supplementary Methods). Videos were taken from both dorsal/ventral (the *xy* plane) and lateral view (the *yz* plane), but the majority of videos (60%) analyzed were from *xy* plane due to difficulty in obtaining video from lateral view.

### Vector field calculation

Videos were imported into DaVis (version 8.2.1, LaVision GmbH) for flow field computation. Prior to cross-correlation calculation, masking of larvae was performed with three background removing algorithms (smoothing, sliding maximum, and sliding minimum subtractions), followed by thresholding. A multi-pass algorithm with a decreasing size of interrogation windows (from 64 × 64 to 32 × 32 pixels for *P. planus*, and 96 × 96 to 64 × 64 pixels for *T. japonica*, both with 50% overlaps) was used in cross-correlation computation on instantaneous flow velocity vectors. The size of interrogation window was chosen based on density of seeding particles such that each window contains a density of >15 particles. Vector post-processing was performed to remove outlier vectors before exporting the final velocity vectors *V* into grids of 80 × 64 cells (each cell represents 16 × 16 pixels, with (u,v) components representing velocity in (x,y) directions) for further calculations. Vector field interpolation was performed for *T. japonica* to achieve the same density of vectors in the final vector fields for both species observed.

### Locomotion: swimming velocity and kinematics

**Fig. 1:**
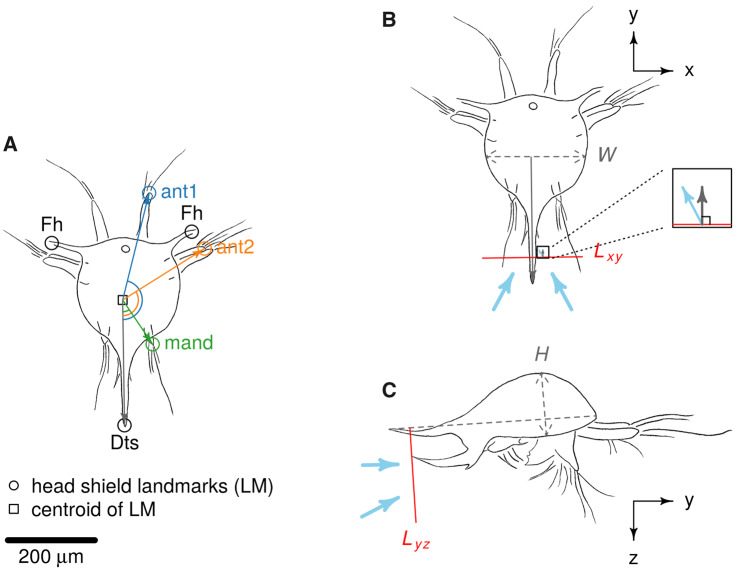
(**a**) Landmark placements (Fh and Dts) for kinematics analysis. Flux line definitions for flux calculations for top view (**B**) and side view (**C**). Flux calculation involves the projection of velocity vector passing the defined line onto normal direction and calculation of the product of the magnitude of the projected vectors and the magnitude of the defined line. *W*, carapace width; *H*, carapace height; *L_xy_*, line for flux calculation for top view with length equal to length of *W*; *L_yz_*, line for flux calculation for lateral view with length equal to 1.5× length of *H*; Fh, frontal horn; Dts, dorsal thoracic spine; ant1, antennule; ant2, antenna; mand, mandible.

For swimming and kinematics analyses, about 40 frames were extracted from each video covering a complete beat cycle sampled at approximately equidistant time points. Identification of beat cycles was first estimated from the videos by eye and later quantitatively determined based on the angular positions of the swimming appendages. Displacement of the swimming nauplius was calculated from the distance between centroids of three body landmarks on the larva between frames. These three body landmarks are tips of frontal horns and the tip of the dorsal thoracic spine (Fig. 1). Direction of displacement was determined by looking at the sign of the dot product of the displacement vector and the vector from centroid to tail spine (dorsal thoracic spine, designated as $\vec{\mathrm{CT}}$ here). A negative sign of the dot product indicates opposite direction with $\vec{\mathrm{CT}}$ and defined as forward displacement and *vice versa*. A value of zero was defined as no displacement in direction parallel to $\vec{\mathrm{CT}}$. Cumulative displacement curves, i.e., the cumulative sums of displacement of naupliar body’s centroid over time, were used to compare displacement patterns of moving naupliar body. Reynolds number was calculated as Re=*UL*/*V*, with *U* the average swimming speed, *L* the larval length, and *V* the kinematic viscosity of seawater at 25°C, 33 psu. To compare the efficiency of propulsion per beat, we calculated the ratio of forward to reverse displacement. Angles of three pairs of naupliar appendages—antennule (ant1), antennae (ant2), and mandible (mand)—were defined as the angle formed between each vector of centroid to appendage tip $\vec{\mathrm{CA}}$ and $\vec{\mathrm{CT}}$, calculated as

$$\theta =\;\cos^{-1}\left(\frac{\vec{\mathrm{CA}}\;\cdot \;\vec{\mathrm{CT}}}{\left\|\vec{\mathrm{CA}}\|\|\vec{\mathrm{CT}}\right\|}\right).$$

Marking of body landmarks and tips of appendages was performed in tpsDIG2 (version 2.30; Rohlf 2010). Angular positions for appendages were digitized only for the right side. Swimming velocity and the angular velocity of the appendages were calculated by taking the time derivative of larval centroid displacement and angular displacement of the appendages, respectively. Two metrics were calculated to quantify the difference in beat timing of appendages: angular separation between combinations of appendages, and proportion of time that combinations of appendages moved in same direction (beating or retracting). These metrics were compared with a permutational *T*-test run with 9999 permutations in R.

### Locomotion: vortex circulation

Swimming nauplii produce vortices with their beating appendages as they propel forward. The vortex structure and strength is related to the amount of thrust produced. We quantified and compared vortex circulation produced by the beating appendages of the nauplii directly from the flow field. Circulation Γ was calculated from the surface integral of vorticity ω for the area A bounded by vortices

Γ= ∬ω⋅dA.

ω was calculated in DaVis software. Discrete approximation of circulation was computed as sum of vorticity at grid position (x, y) multiplied by the area represented by the cell for each frame at time *t*

$$\Gamma =\sum \omega \;\left(x,y,t\right)\;dxdy.$$

Only vortices at the right side of the larva were used for calculation and the vortices were compared between species with a permutational *T*-test run with 9999 permutations in R.

### Predation risk: spatial attenuation of flow

Flow disturbance generated by nauplii is expected to decay over distance, and a faster spatial decay imposes less risk of being detected by a potential predator (Kiørboe et al. 2014). Flow speed $\left\|V\right\|$ is a function of distance from the larva *r*

$$\|V\|\propto r^{n}.$$

To compare the risk of predation presented as magnitude of hydrodynamic signal, we calculated *n*, the power for spatial attenuation from the velocity field. The computation was performed with a method similar to that of Kiørboe et al. (2014), where binning of flow speed was first performed with different thresholds of speed U*. Distance of the spatial extent of the flow was then determined as radius *r* of a circle of area equivalent to the area covered by the binned speed *S* (U*). The power *n* was estimated by a power law fitting, i.e., by regression analysis with $\ln\left(U^{*}\right)$ and ln⁡(r) as *y* and *x* of the regression equation, respectively. Power *n* was then obtained from the slope of the regression fit. We compared the power of spatial attenuation of flow at the peak of the power stroke between the two species with a *T*-test.

### Feeding: flux

We calculated flux toward the food capturing region (vicinity of labrum) of a nauplius during the recovery stroke to compare the volume of feeding current generated by the nauplii. *Polyascus* nauplii do not feed and possess only a vestigial labrum; thus, the water flux represents a hypothetical equivalent to *Tetraclita* nauplus’s feeding current. In a three-dimensional (3-D) velocity field, flux *Φ* can be calculated as the surface integration of velocity vectors passing through a defined area *A* at an angle normal to the surface

$$\Phi_{x,y,z}=\iint (u,\;v,w)\cdot \hat{n}\;dA,$$

where *w* is the velocity in *z*-direction, $\hat{n}$ is the normal unit vector, and the dot product gives the magnitude of velocity vector projected onto the normal direction. Since our PIV data are only cross-sectional (2-D), we computed “flux” passing through a line segment of length equal to body width of the larva from the 2-D vector field (Fig. 1). A discrete approximation was computed by summing up the magnitude of the velocity vectors projected on normal direction multiplied by the length represented by each velocity vector

$$\Phi_{x,y}=\sum \left(u,v\right)\cdot \;\hat{n}\;dl.$$

A similar computation was performed for velocity fields in lateral view $\left(v,\;w\right)$ for line segments of length equal to 1.5× body height of a larva in the ventral direction (Fig. 1). Fluxes were calculated from both the earthbound frame of reference (defined as “absolute flux”) and in the nauplius’ frame of reference (defined as “relative flux,” which is the absolute flux minus the naupliar body’s velocity). In other words, relative flux estimates flow relative to the position of the nauplius’ body, which is essential to determine whether flow carrying potential food particles is approaching or leaving the food capturing area. Relative fluxes in both top and lateral views were compared between species with a *T*-test.

## Results

### Swimming proficiency and efficiency

**Fig. 2:**
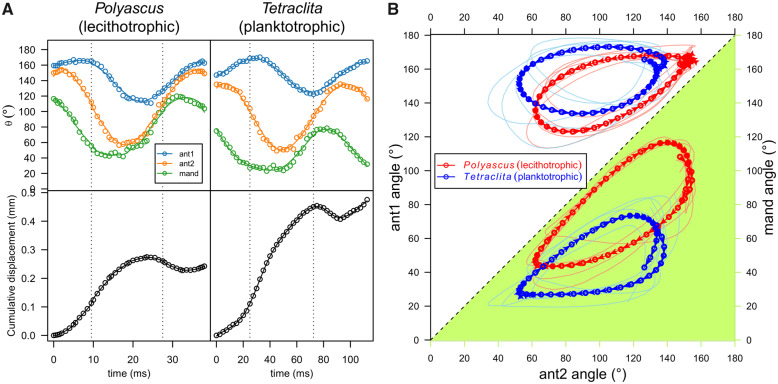
(**a**) Representative profiles of angular positions of the appendages and cumulative displacement of the naupliar body’s centroid over a stroke cycle. Dashed lines indicate mid-power and mid-recovery stroke, defined as frames with highest angular speeds for ant2 during power stroke and recovery strokes, respectively. Profiles of other individuals observed are presented in Supplementary Fig. S1. (**B**) Lissajous curves of the angular positions of pairs of appendages. Lower diagonal (green shaded labeled with green axes): antenna-mandible (ant2-mand); upper diagonal: antenna-antennule (ant2-ant1). Thin, light lines represent curves of different individuals while thick, dark lines are the means of these curves for two different species.

The non-feeding nauplii of *Polyascus* swam more than twice as fast as the feeding *Tetraclita* nauplii at ∼29 body length s^−1^ (7.7 ± 0.4 mms^−1^) compared with ∼10 body length s^−1^ (4.5 ± 0.5 mms^−1^) (Table 1). The higher swimming speed of *Polyascus* nauplii put these smaller nauplii (265.0 ± 7.4 µm carapace length) in similar Reynolds number (ca. 2) with the larger (447.4 ± 16.8 µm carapace length) *Tetraclita* naplii (Table 1). High speed videos of swimming nauplii (Supplementary Video S1) showed that both the fast and slow swimmers suffered from backward displacement during the recovery stroke. In fact, *Tetraclita* nauplii pushed themselves back less during the recovery stroke relative to forward displacement during the power stroke, making them more efficient in terms of the forward:backward displacement ratio (Fig. 2A and Table 1).

### Swimming kinematics

The swimming velocity difference is best explained by the large difference in beat frequency between the species. *Polyascus* nauplii beat their appendages at frequencies approximately three times that of *Tetraclita* nauplii (Table 1), which translates into higher angular speeds in all pairs of appendages (Table 1). In addition, *Polyascus* nauplii beat their mandibles at larger amplitudes (Fig. 2A, Table 1, and Supplementary Videos S2 and S3). There was no significant difference in the beat amplitude for the other two pairs of appendages (Table 1). For both species, antennae beat with the largest amplitude and antennules beat with the smallest. Within each species, there was no significant difference in angular speeds between power and recovery strokes, except for the mandibles of *Polyascus* nauplii (Table 1).

Besides differences in frequency and amplitudes, the two species had distinctive phase shift patterns between pairs of appendages, summarized in Lissajous curves (Fig. 2B) and in percentage of appendage pairs moving in the same direction (Table 1). *Polyascus* nauplii swam with a metachronal wave of power strokes that began with mandibles and ended with antennules. This initial movement was followed by a synchronous recovery stroke, during which all pairs of appendages retracted with little separation (Fig. 2). In contrast, *Tetraclita* nauplii swam with only mandibles and antennae beating in a similar metachronal power stroke, but had their antennules moving away from the other two appendage pairs, as evident from the large angular separations at mid power stroke (Table 1). At mid recovery stroke, antennules and mandibles began to move away from each other, enlarging angular separation during *Tetraclita* nauplii’s recovery stroke. In sum, kinematics differences between species were more pronounced during recovery stroke than power stroke.

### Vortex circulation

**Fig. 3:**
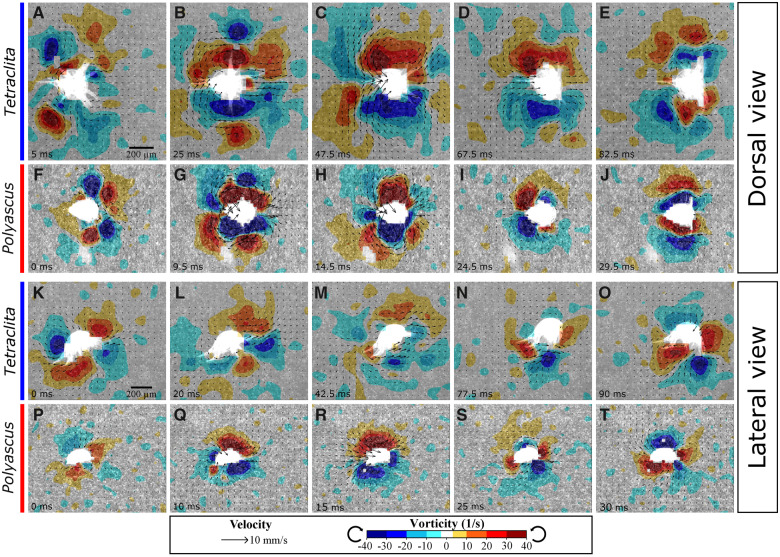
Snapshots of combined velocity and vorticity fields around swimming nauplii. (**A–E**) Dorsal view for planktotrophic *Tetraclita* nauplius. (**F–J**) Dorsal view for lecithotrophic *Polyascus* nauplius. (**K–O**) Lateral view for planktotrophic *Tetraclita* nauplius. (**P–T**) Lateral view for lecithotrophic *Polyascus* nauplius. Time stamps correspond to time axis in Fig. 2A. Both species use the same scale bars. Animations are shown in Supplementary Videos S4 and S5.

Differences in kinematics were also reflected in differences in vorticity circulation. Strokes of *Polyascus* nauplii created higher vorticity (*ω*) than strokes of *Tetraclita* nauplii. Vorticity (ω) at the end of the power stroke was −52.6 ± 5.3 s^−1^ (SE) compared with −24.2 ± 0.6 s^−1^, which corresponded to the higher angular speed of beat (Table 1). However, the vortex circulation of *Polyascus* nauplii had on average 46% smaller spatial extent than that of *Tetraclita* nauplii (Fig. 3). Thus, when integrated over area, the circulation of the body vortices (Γ) was of similar magnitudes between the two species at the end of power stroke (Table 1 and Supplementary Fig. S3). However, the relative contribution of each limb toward vorticity circulation differed qualitatively between the two species (Fig. 3 and Supplementary Videos S4 and S5). Body vortices created by mandibles’ beat in *Tetraclita* nauplii were at more posterior positions and had a smaller extent, which corresponded to a smaller amplitude of beat of the mandibles in *Tetraclita* than in *Polyascus* nauplii (Fig. 3A, F). In addition, the extent and magnitude of vorticity created by mandibles’ beat was considerably smaller than that by antennae in *Tetraclita* nauplii, corresponding to the large difference of amplitude between these two pairs of appendages (Fig. 3A, B). In contrast, *Polyascus* nauplii’s mandibles and antennae created vortices of similar extent and magnitude during power strokes (Fig. 3G, H).

### Spatial attenuation of fluid disturbance

**Fig. 4:**
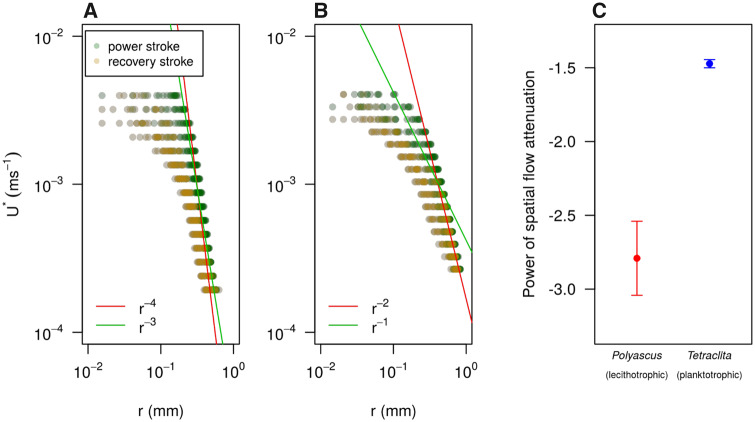
Representative profile of U* plotted against *r* for lecithotrophic *Polyascus* nauplius (**A**) and planktotrophic *Tetraclita* nauplius (**B**), where U* is the binned flow speed and *r* is radius of the circle with equivalent area to the area occupied by U* in the flow field. Power fitting lines for idealized models of spatial attenuation of flow at the peak of power stroke (correspond to the right most data points in dark green) are shown. (**C**) Comparison of empirical power fittings for spatial flow attenuation at the peak of power stroke summarized as mean±SE, with *n *=* *5 for each species. Mean difference is significant *P* < 0.01. Profile of U* plotted against *r* and time evolution of flow attenuation power for all individuals are presented in Supplementary Figs. S4 and S5, respectively.

*Polyascus* nauplii swam with a small area of influence (with flow ≥ 0.0005 ms^−1^, Kiørboe et al. [2014]) at the end of the power stroke, around half of that of *Tetraclita* nauplii (Table 1). Area of influence varied through the beat cycle, but the observed differences between species are robust (Supplementary Fig. S2). This difference can be explained by faster spatial flow attenuation observed for *Polyascus* nauplii (Fig. 4). At the peak of the power stroke, flow speed near the *Polyascus* nauplii body was higher, but it attenuated sharply with distance with an average power of −2.79 compared with −1.47 in *Tetraclita* nauplii (Fig. 4C). This sharp decline in flow speed limited the spatial extension of fluid disturbance created, allowing the non-feeding *Polyascus* nauplii to swim more quietly.

### Flux and feeding current

From velocity fields, potential paths of fluid flow carrying food particles toward the nauplius body could be observed. During the power stroke, fluid was pushed toward the body of the nauplius from both left and right sides toward its posterior end (Fig. 3). During the recovery stroke, fluid was pulled from the posterior end toward the body by the appendages, creating a suction feeding current toward the food capturing region.

Relative flux, calculated from flow relative to the moving body of the nauplius, shows that fluid did not flow toward the nauplius’ body during the power stroke; instead, fluid followed the moving body of the nauplius, going forward due to viscosity (see Supplementary Fig. S6 for absolute flux and Supplementary Fig. S7 for relative flux; and flux calculated from lateral views in Supplementary Figs. S8 and S9). Because the velocity of the moving nauplius’s body was about an order of magnitude larger than the fluid flow velocity created by the swimming stroke, relative velocity of flow toward the body’s proximity was dictated by body velocity calculated from the centroids of body landmarks (Supplementary Fig. S10). Thus, flux toward the body was achieved only during the recovery stroke, when body velocity was reversed.

**Fig. 5:**
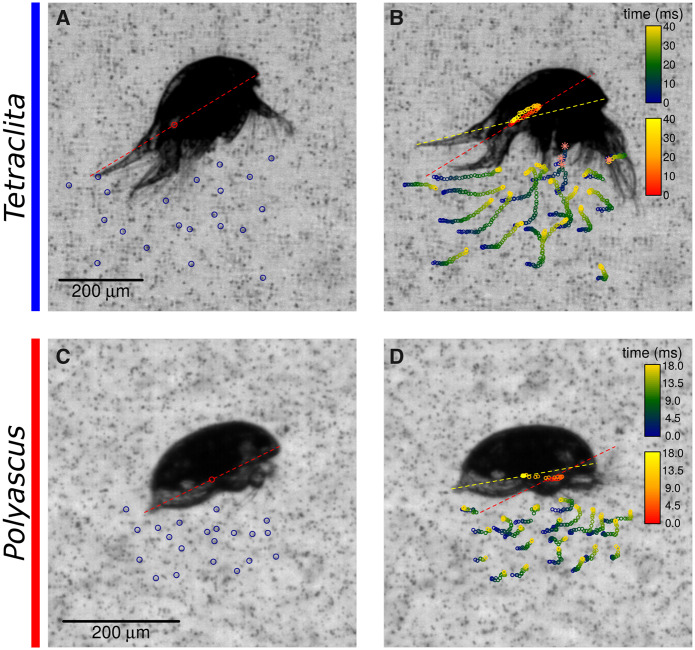
Particle tracking illustrating suction current generated during the recovery stroke. Centers of body axis were traced from the beginning (red-dashed line, **A**, **C**) and end of the recovery stroke (yellow-dashed line). The path of particles on the ventral side of nauplii (blue, green, and yellow dots) were traced over the duration of the recovery stroke for the planktotrophic *Tetraclita* (**A**, **B**) and the lecithotrophic *Polyascus* (**C**, **D**) nauplius, respectively. Asterisks indicate frames at which the particles could no longer be traced. Particle tracing animation is shown in Supplementary Video S6.

Relative fluxes were not significantly different between the two species (Table 1) and *Polyascus* nauplii could bring particles to the proximity of their body easily with a backward movement during the recovery stroke, even though they did not need to feed. Because the transport of particles could not be followed in the Eulerian approach of PIV, we analyzed the particle path by simply tracking particles individually to investigate their fates. From the tracing of particles during the recovery stroke (Fig. 5 and Supplementary Video S6), it was revealed that the *Tetraclita* nauplius drew particles toward its food capture area under the labrum with good accuracy, i.e., the end of the particle paths matched with the capture region at the end of recovery stroke. Suction current was also generated during the recovery stroke for *Polyascus* nauplii, but was not directed toward the vestigial labrum.

## Discussion

The observed planktotrophic and lecithotrophic barnacle nauplii differed in locomotory performance, generation of fluid signal, and manipulation of near-body fluid flow. The integrated process of feeding and swimming observed in *Tetraclita* nauplii led to compromises in swimming speed and predation avoidance. *Polyascus* nauplii, which are released from the need of feeding, swim fast with rapid fluid disturbance attenuation. This unique comparison of “swimmer versus feeder” reinforces the importance of hydrodynamics in shaping predation risk, and thus, zooplankton evolution (Kiørboe et al. 2014). Linking swimming kinematics and hydrodynamic consequences of contrasting larval forms also helps improve our mechanistic understanding of how functional needs shape the evolution of naupliar morphology.

### Optimal propulsion of lecithotrophic nauplius larvae

The swimming speed of the *Polyascus* nauplii is the fastest recorded for barnacles thus far in terms of both body length and distance per unit time (compared with Walker [2004]). Given that nauplii of both species had similar Reynolds number (ca. 2), inertial effect contributed little to *Polyascus* nauplii’s faster swimming speed than *Tetraclita* nauplii. There are two possible mechanisms that contribute toward this fast swimming of the non-feeding larvae, namely high beat frequency and synchronized beat pattern. Despite the circulation (Γ) being similar between the two species observed, the *Polyascus* nauplii complete triple the amount of beat cycles within a unit time, and hence, traverse a larger distance. Furthermore, swimming of nauplii of *Polyascus* resembled the “swimming-by-jumping” observed in copepod nauplii in which metachronal stroke was used (Borg et al. 2012). Metachronal stroke, featured with appendages in sequential power strokes and simultaneous recovery strokes, has been identified as the most efficient swimming mechanism for multi-legged swimmers (Lenz et al. 2015; Takagi 2015). Other similarities, such as higher frequency of appendage beat and higher stroke amplitude for mandibles (Borg et al. 2012), were also observed in *Polyascus* nauplii. These shared characteristics likely help increase swimming speed, promoting convergence to metachronal stroke among fast swimming nauplii.

### Tradeoffs between feeding and efficient swimming

In contrast to fast swimming nauplii, planktonic crustacean nauplii that cruise slowly through the water do not share a single stroke pattern (Moyse 1984; Johnson et al. 2011; Borg et al. 2012). While the antennae are the main appendage for propulsion, the roles of the remaining two pairs of limbs vary (Gauld 1959; Walker et al. 1987; Anderson 1993; Williams 1994b). In *Tetraclita* nauplii, antennules moved in anti-phase to antennae and mandibles for a large proportion of time. This observation supports the previous view that barnacle nauplii’s antennules contribute little to propulsion (Walker et al. 1987).

In fact, this anti-phase beating of antennules might have a role in “anchoring” the moving body of the nauplius during recovery stroke. Our particle tracking comparison suggested that successful capture of particles in planktotrophic barnacle nauplii depends on matching between particles brought by the suction current produced by the antennae and mandibles and the position of the feeding chamber at the end of the recovery stroke. Excessive backward displacement of the nauplius body in any direction could shift the focus of the suction flow, resulting in a misdirected flow. Therefore, retarded backward displacement during the recovery stroke, i.e., the “anchoring effect,” could be crucial in “guiding” the feeding current. The observed anti-phase beating ensured that antennules were fully extended when antennae reached the peak of retraction speed. Together with drag increasing long frontal horns and tail spines, the spanning antennules could contribute toward the anchoring effect for *Tetraclita* nauplii. However, such backward displacement dampeners likely come at the cost of propulsion as they add burden to forward displacement during the power stroke.

The observed mechanism for reducing backward displacement is different from that suggested for copepod nauplii, which involves the movement of mandibles (Borg et al. 2012). *Tetraclita* nauplii’s mandibles beat with small amplitude. The limited radial motion of mandibles is likely a result of their known direct role in pushing food particles toward the food collecting region with their medially directed setae (Gauld 1959; Anderson 1993). Supporting this notion, the contrasting mandible beat pattern between the feeding and non-feeding nauplii did correspond to differences in circulation (Fig. 3). These observations highlight that feeding imposes functional constraints on kinematics such that movement patterns favoring efficient propulsion do not coincide with those for effective particle capture. The resulting diversity of kinematics in turn help shape diversity of naupliar body forms (Wong et al. 2018).

### Tradeoffs between feeding and predation risk

Good feeders are often associated with poor swimming (Strathmann and Grünbaum 2006). But the feeding process not only compromises swimming performance, it also puts the feeding nauplii at risk of predation due to the greater fluid signal generated (Kiørboe et al. 2014). Fast swimmers characterized by a short power stroke duration relative to the viscous time scale generate a fluid flow that attenuates quickly, which is well studied in copepod adults (Jiang and Kiørboe 2011). Nauplii of neither copepods nor barnacles could swim as hydrodynamically quietly as the copepod adults. Nonetheless, reduced fluid signal is evident in these fast-swimming crustacean nauplii. Spatial attenuation power is similar in copepod and barnacle nauplii: ∼*r*^−3^ for *Polyascus* and jumping copepod nauplii, and ∼*r*^−1.5^ for *Tetraclita* and cruising copepod nauplii (Fig. 4). This observation again highlights how common limiting factors (biomechanical constraint from naupliar body plan) and driving forces (selection pressure from predation and starvation risk) shape hydrodynamics of larval locomotion.

### Planktotrophy versus lecithotrophy

The better performance in locomotion and predation avoidance in lecithotrophic nauplii prompts us to re-visit the question of why loss of feeding is not more common (Strathmann 2018). One possible explanation is that lecithotrophy is costly in terms of parental investment in eggs. *Polyascus* and other rhizhocephalan barnacles are parasites that have plenty of nutrients at their disposal (Høeg 1995), removing the penalty of investment. The other possibility is that planktotrophy confers benefits: nauplii can spend longer times for dispersal and accumulate energy storage to increase chances of post-settlement survival. Such long-distance dispersal ability, though disputed (Strathmann 2018), could be essential for population maintenance of sessile barnacles.

Our kinematic and hydrodynamic comparisons connect morphological differences among barnacle nauplii to their contrasting ecological needs. The globular-shaped lecithotrophic nauplii swam faster with metachronal limb beats and were hydrodynamically quietly. In contrast, the planktotrophic nauplii increased drag (through anti-phase limb beat and body extensions) to create an accurately-directed feeding current. Thus, the functional trade-offs between feeding, locomotion, and predator avoidance impose kinematic and hydrodynamic constraints, which in turn help shape the evolution of larval form.

## Author contributions

J.Y.W. collected the data and carried out the analysis. All authors conceived and designed the study, drafted the manuscript, and gave approval for publication.

## Data accessibility

Data and codes to reproduce the results are deposited in Open Science Framework (https://osf.io/r9abn/) and Github (http://github.com/jinyung/npiv).

## Supplementary Material

obaa011_Supplementary_Data

## List of symbols

$\vec{\mathrm{CA}}$: Vector from body centroid to appendage
$\vec{\mathrm{CT}}$: Vector from body centroid to tail spine
θ: Angle formed between $\vec{\mathrm{CA}}$and $\vec{\mathrm{CT}}.$
V: Velocity vector
*u, v, w*: Velocity vector components in *x*, *y*, and *z* directions
u: Velocity field on *xy* plane
*U**: Speed of velocity vector exceeding a threshold
*r*: Distance from larva, radius of circle with equivalent area occupied by *U**
Φ: Flux
Γ: Circulation
ω: Vorticity
