# Supplementary material for: Evolution of Feeding Shapes Swimming Kinematics of Barnacle Naupliar Larvae: A Comparison between Trophic Modes
Source: Integr Org Biol. 2020 Apr 17;2(1):obaa011. doi: 10.1093/iob/obaa011 (PMC7671147; doi:10.1093/iob/obaa011)
Supplement: obaa011_Supplementary_Data [file obaa011_supplementary_data.docx]

# Supplementary Methods

## Depth of field estimation

We used two approaches to estimate depth of field. The first approach [1] calculates near distance of acceptable sharpness (*D_n_*) and far distance of acceptable sharpness (*D_f_*) based on hyperfocal distance (*H*):

$$H=\frac{f^{2}}{NC}+f$$

$$D_{n}=\frac{s\left( H-f \right)}{H+s-2f}$$

$$D_{f}=\frac{s\left( H-f \right)}{H-s}$$

Where *f* = lens focal length; *s* = focal distance; *N* = f-number and *C* = circle of confusion. The focal distance *s* was defined as the distance from object to the principal plane of the lens (= working distance + distance of principal plane from the front end of the lens body). Since we did not know the exact position of the principal plane inside the lens body (with a length of ca. 90mm), a rough estimate was used (*s* = 70 – 80mm). We used the pixel size on the camera sensor (10µm) for value of *C*. Then, depth of field (*T*) is calculated as:

$$T=D_{f}-D_{n}$$

An estimate of 31-71 µm of *T* was obtained for this approach.

The second approach [2] estimates *T* based on the magnification (*m*) of image. With the knowledge of the sensor pixel size and the actual size of the nauplius, we calculated *m* from the ratio of image size on camera sensor to object size. *T* was calculated as:

$$T=\frac{2CN\left( 1+m \right)}{m^{2}}$$

An estimate of 30-35 µm of *T* was obtained for this approach.

# Supplementary References

[1] Greenleaf, AR. 1950 *Photographic optics*. New York: The MacMillan Company. p.25 – 27.

[2] Jacobson R, Ray SF, Attridge GG, Axford NR. 2000 *The manual of photography: photrographic and digital imaging*. 9th ed. Woburn (MA): Focal Press.

# Supplementary Figures


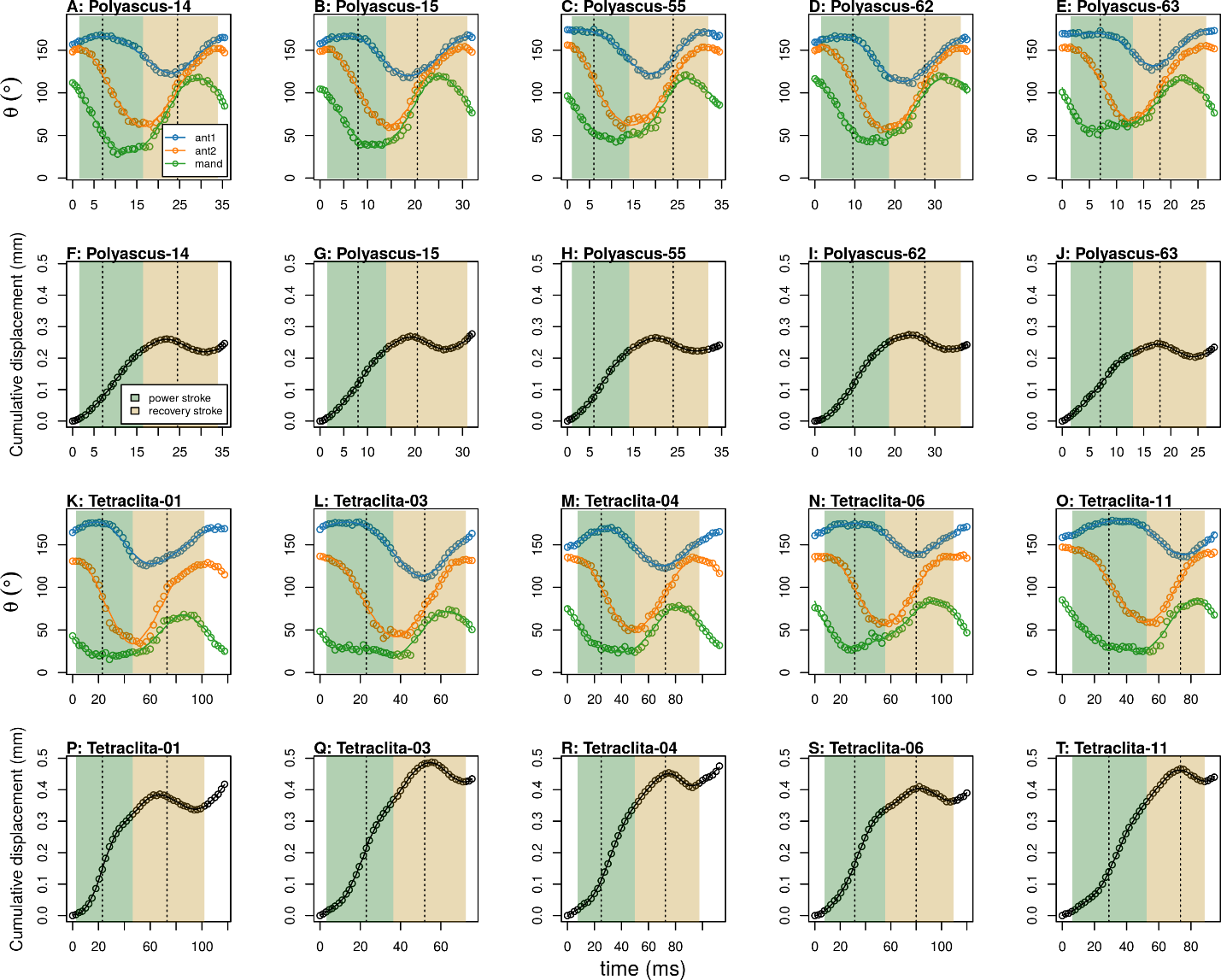


**Figure S1**: Time evolution of angular positions of the appendages (A-E, K-O) and cumulative displacement (F-J, Q-T) over a stroke cycle for all individuals observed (A-J: lecithotrophic *Polyascus* nauplii; K-T: planktotrophic *Tetraclita* nauplii; Representative individuals presented in Figure 2 and 3A-J were from video ID “Polyascus-62” and “Tetraclita-04”). Dashed lines indicate mid-power and mid-recovery stroke, as in Figure 2. Shadings separate power and recovery strokes (defined as the maximum to minimum angle, and minimum to maximum angle of antennule, respectively).

**
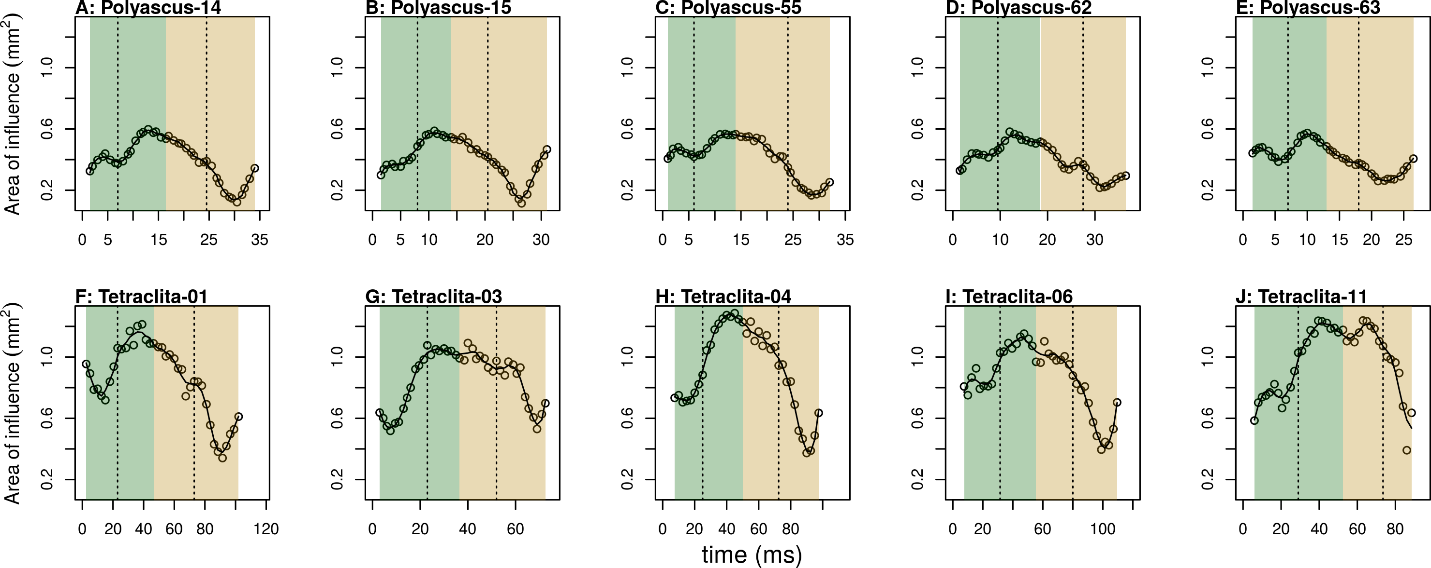
**

**Figure S2**: Time evolution of area of influence for flow velocity > 0.0005ms^-1^ for all individuals (A-J: lecithotrophic *Polyascus* nauplii; K-T: planktotrophic *Tetraclita* nauplii). Refer to Figure S1 for meaning of dashed lines and shadings.

**
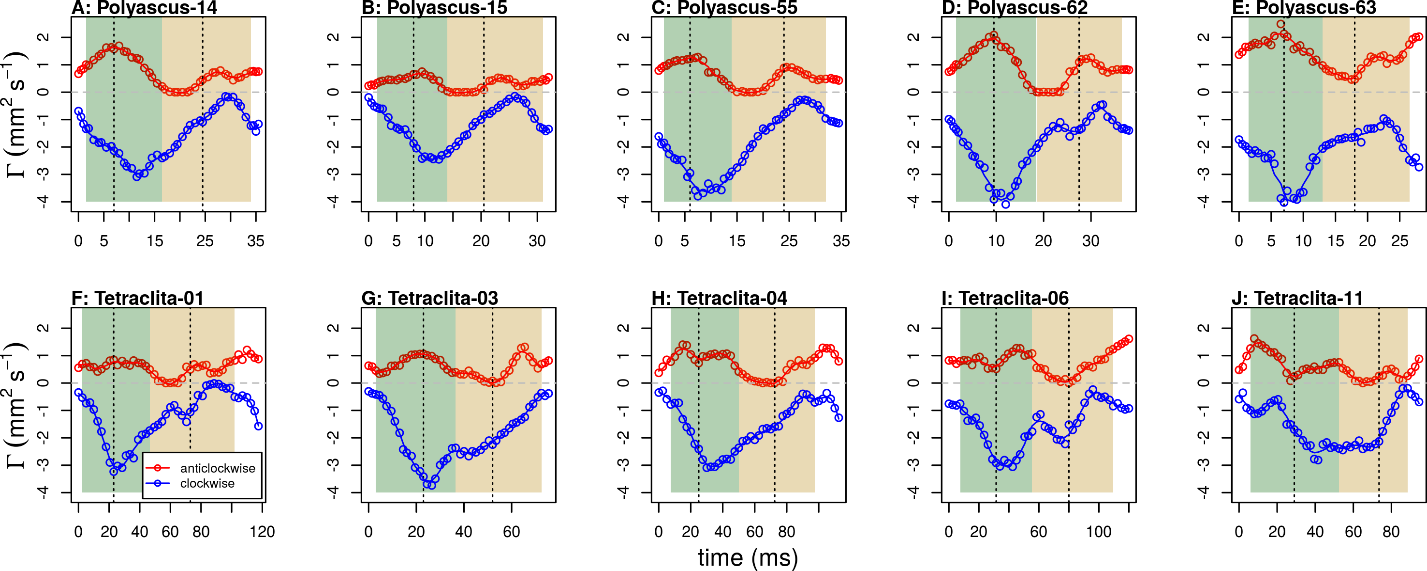
**

**Figure S3**: Time evolution of circulation for all individuals (A-E: lecithotrophic *Polyascus* nauplii; F-J: planktotrophic *Tetraclita* nauplii). Only circulation from right side of the body of the nauplii was included in the analysis. Refer to Figure S1 for meaning of dashed lines and shadings.


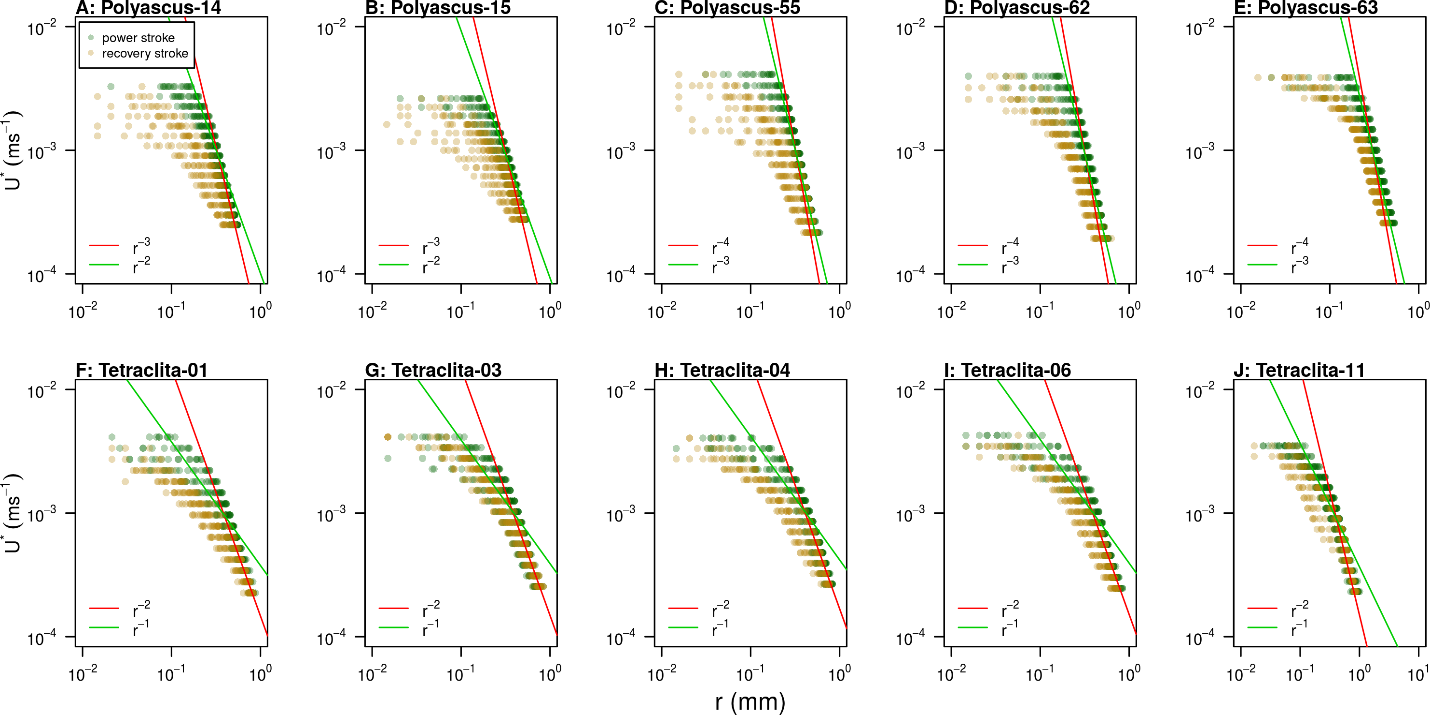


**Figure S4**: Profiles of *U** plotted against *r* for all individuals (A-E: lecithotrophic *Polyascus* nauplii; F-J: planktotrophic *Tetraclita* nauplii).


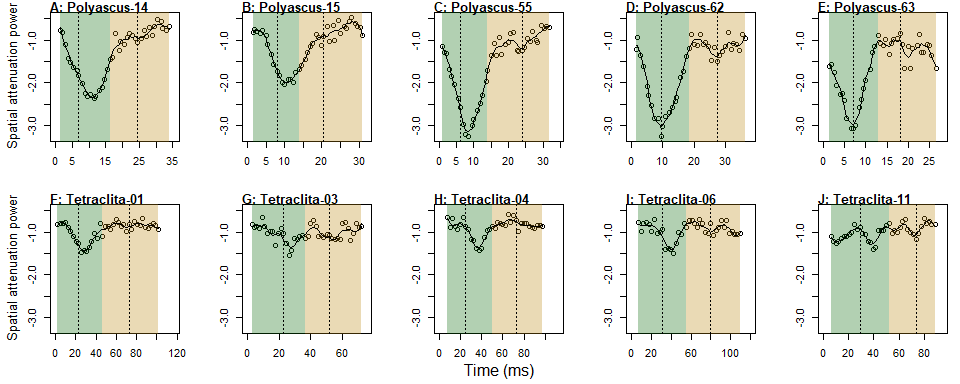


**Figure S5**: Time evolution of spatial attenuation power for all individuals (A-E: lecithotrophic *Polyascus* nauplii; F-J: planktotrophic *Tetraclita* nauplii). Refer to Figure S1 for meaning of dashed lines and shadings.


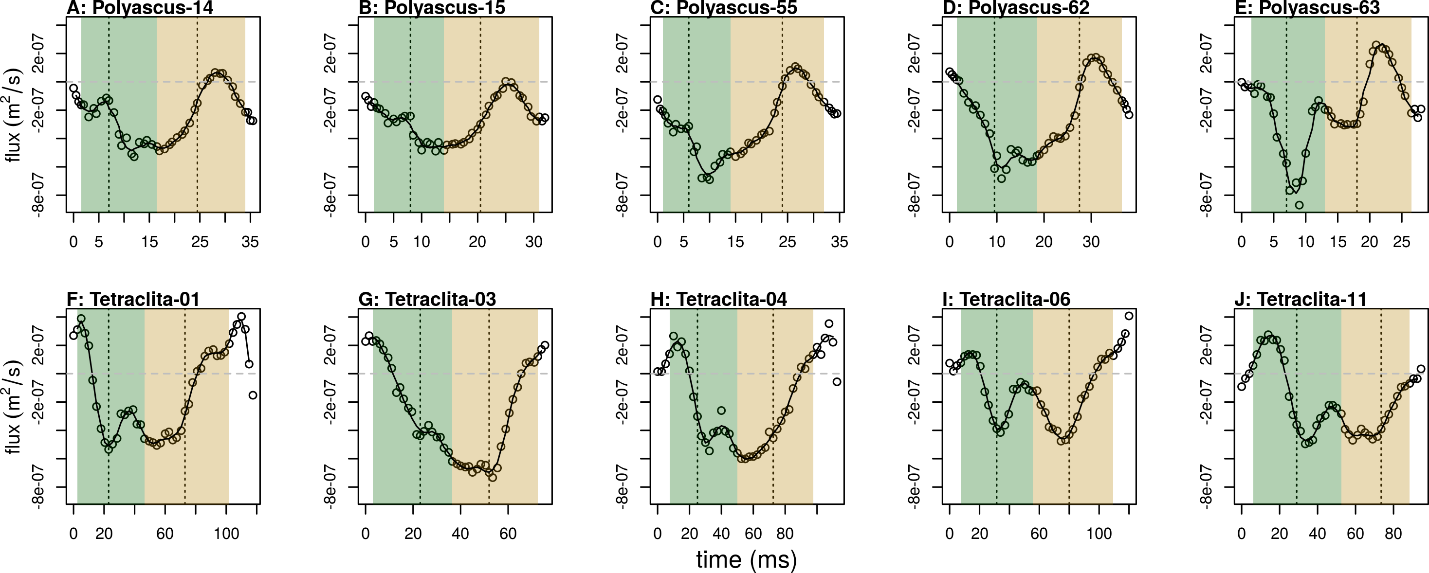


**Figure S6**: Absolute flux from dorsal/ ventral view for all individuals (A-E: lecithotrophic *Polyascus* nauplii; F-J: planktotrophic *Tetraclita* nauplii). Flux values were calculated using earthbound frame of reference. Horizontal dashed line = zero. Refer to Figure S1 for meaning of vertical dashed lines and shadings.


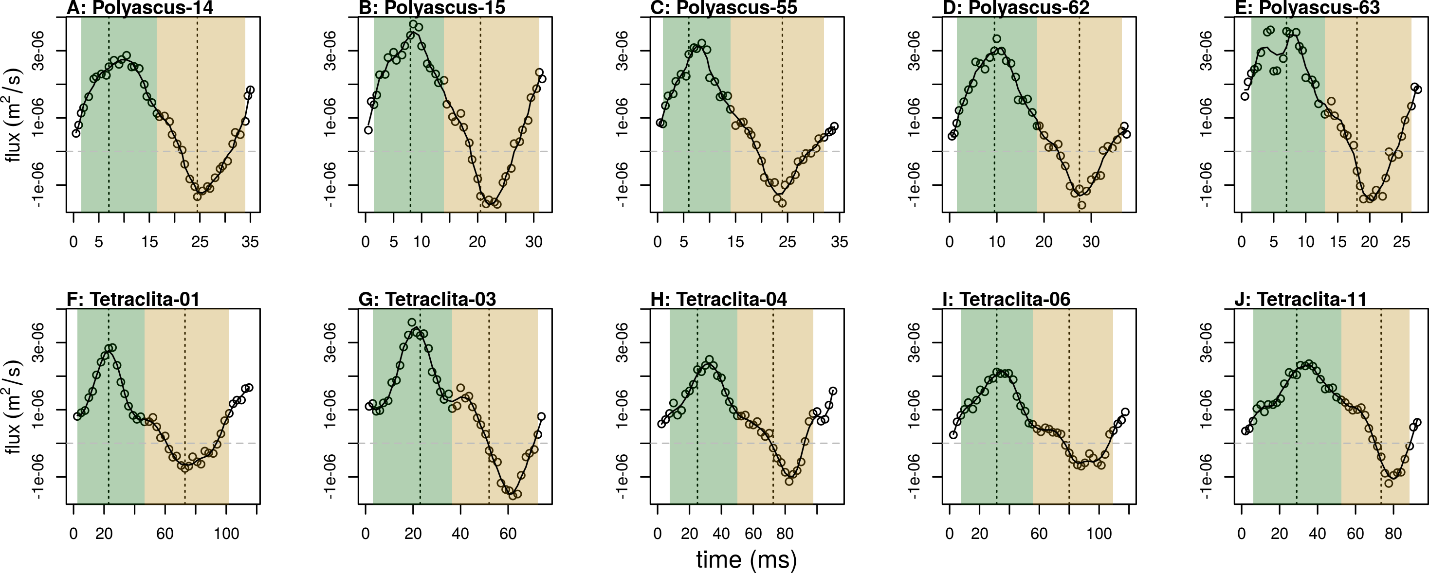


**Figure S7**: Relative flux from dorsal/ ventral view for all individuals (A-E: lecithotrophic *Polyascus* nauplii; F-J: planktotrophic *Tetraclita* nauplii). Flux values were calculated using nauplius larva as the frame of reference. Horizontal dashed line = zero. Refer to Figure S1 for meaning of vertical dashed lines and shadings.


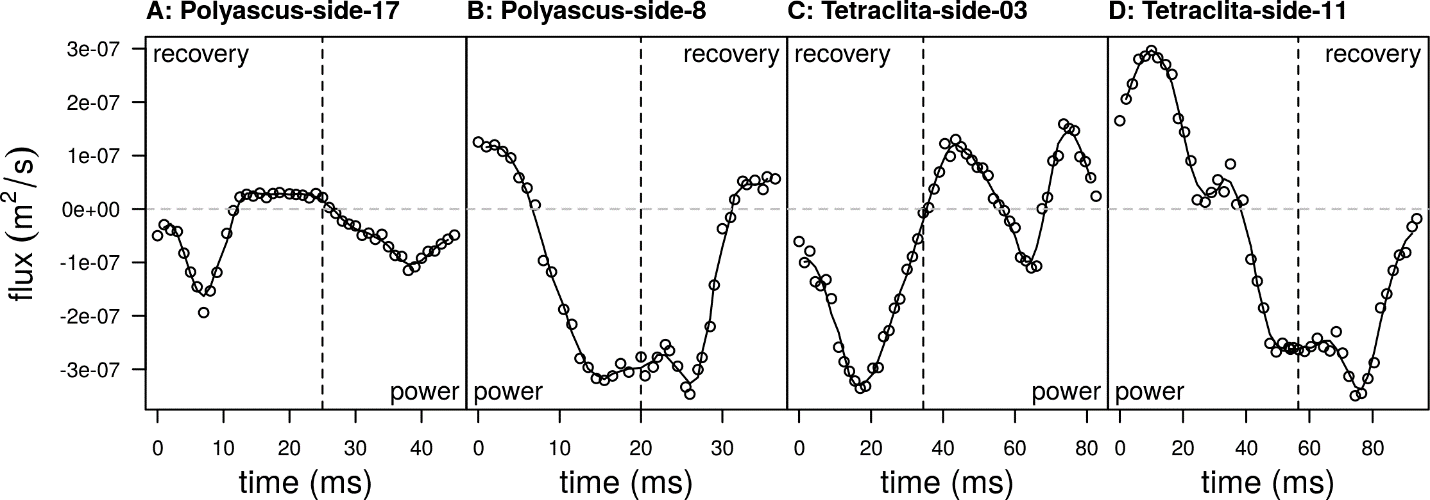


**Figure S8:** Absolute flux from lateral view for all individuals (A-B: lecithotrophic *Polyascus* nauplii; C-D: planktotrophic *Tetraclita* nauplii; Representative individuals presented in Figure 6 and 7 were from video ID “Polyascus-side-8” and “Tetraclita-side-11”). Flux values were calculated using earthbound frame of reference. Vertical dashed line separates power and recovery strokes. Note that order of strokes was different for each individual and was labeled with “power” and “recovery” for power stroke and recovery stroke, respectively.


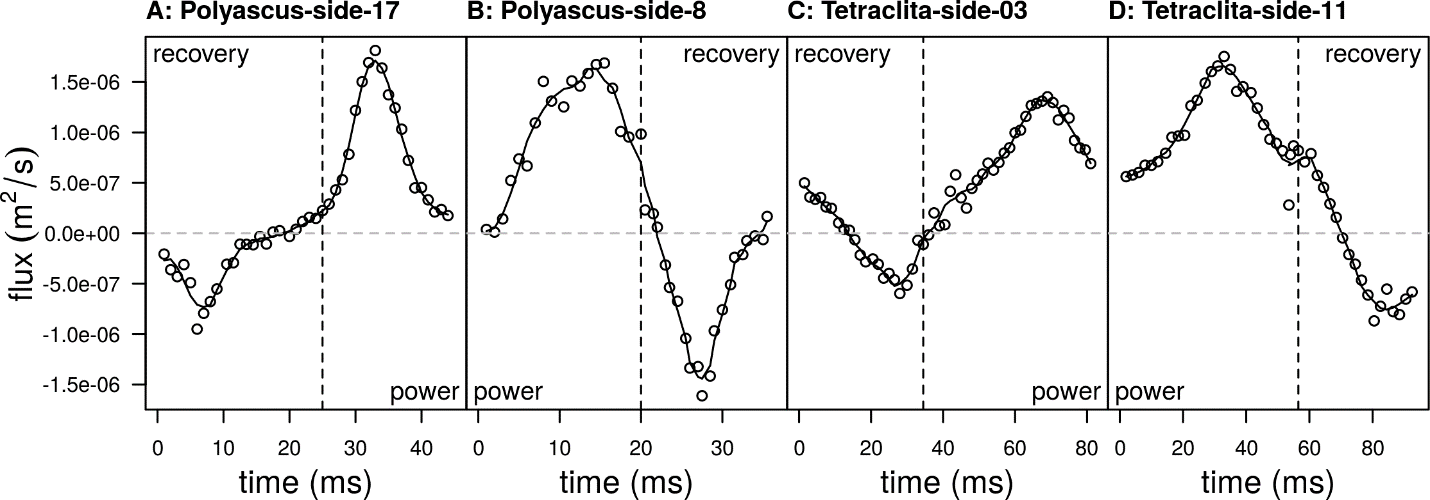


**Figure S9:** Relative flux from lateral view for all individuals (A-B: lecithotrophic *Polyascus* nauplii; C-D: planktotrophic *Tetraclita* nauplii). Flux values were calculated using nauplius larva as the frame of reference. Vertical dashed line separates power and recovery strokes. Note that order of strokes was different for each individual and was labeled with “power” and “recovery” for power stroke and recovery stroke, respectively.


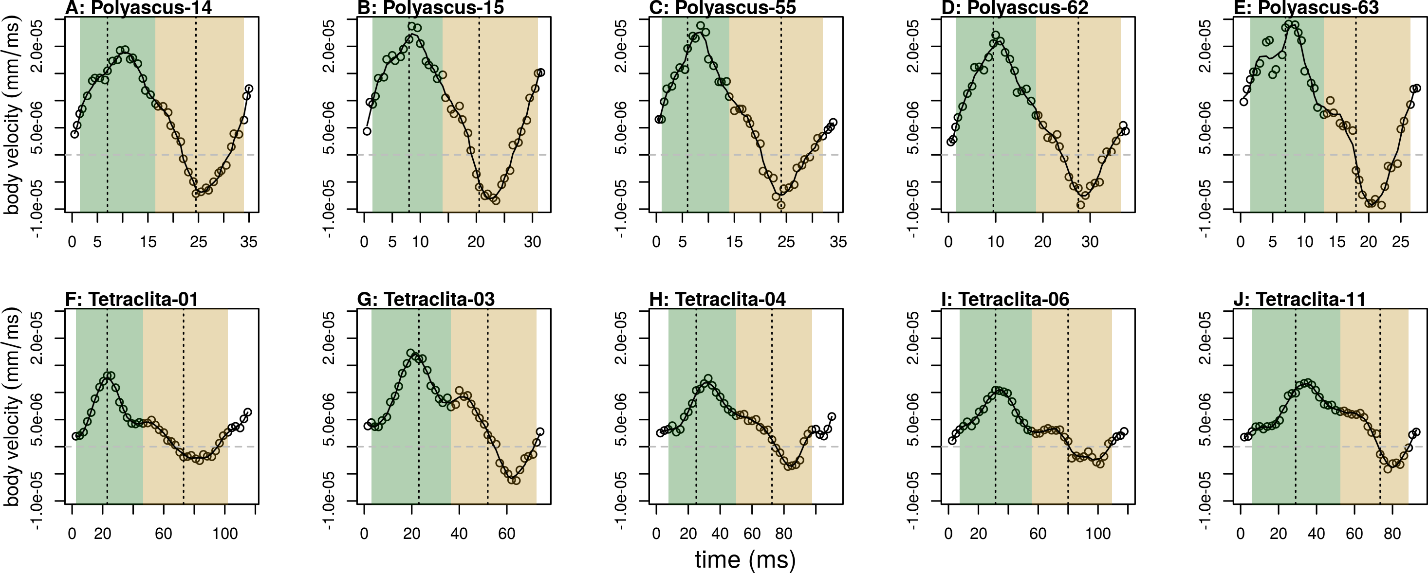


**Figure S10**: Time evolution of nauplius swimming velocity (A-E: lecithotrophic *Polyascus* nauplii; F-J: planktotrophic *Tetraclita* nauplii). Horizontal dashed line = zero. Refer to Figure S1 for meaning of vertical dashed lines and shadings.

# Supplementary Videos

**Video S1**: High speed video of swimming barnacle nauplii at 100× slow motion. Top, nauplius of common intertidal barnacle, *Tetraclita japonica*; bottom, nauplius of the parasitic Rhizocephalan barnacle, *Polyascus planus*. Available at: [https://osf.io/548qt/](https://osf.io/548qt/?view_only=e0864ea8d27546648337bf5cfca966cc)

**Video S2**: Kinematics analysis on appendage movements of the planktotrophic *Tetraclita* nauplius. Refer to Figure 2A for legends. Available at: [https://osf.io/nqr7s/](https://osf.io/nqr7s/?view_only=e0864ea8d27546648337bf5cfca966cc)

**Video S3**: Kinematics analysis on appendage movements of the lecithotrophic *Polyascus* nauplius. Refer to Figure 2A for legends. Available at: [https://osf.io/ktw6x/](https://osf.io/ktw6x/?view_only=e0864ea8d27546648337bf5cfca966cc)

**Video S4**: Velocity and vorticity fields around swimming nauplii from dorsal view (100× slow motion). Refer to Figure 3 for legends. Available at: [https://osf.io/m952x/](https://osf.io/m952x/?view_only=e0864ea8d27546648337bf5cfca966cc)

**Video S5**: Velocity and vorticity fields around swimming nauplii from lateral view (100× slow motion). Refer to Figure 3 for legends. Available at: [https://osf.io/nfh2b/](https://osf.io/nfh2b/?view_only=e0864ea8d27546648337bf5cfca966cc)

**Video S6**: Animation of particle tracking during recovery strokes from side view (200× slow motion). Time labels follow that of Figure 3K-O for *Tetraclita* and Figure 3P-T for *Polyascus* nauplii. Refer to Figure 5 for legends. Available at: [https://osf.io/z3chv/](https://osf.io/z3chv/?view_only=e0864ea8d27546648337bf5cfca966cc)
